# Roles of the prefrontal cortex in learning to time the onset of pre-existing motor programs

**DOI:** 10.1371/journal.pone.0241562

**Published:** 2020-11-09

**Authors:** Beom-Chan Lee, Jongkwan Choi, Bernard J. Martin

**Affiliations:** 1 Department of Health and Human Performance, University of Houston, Houston, TX, United States of America; 2 OBELAB Inc., Seoul, South Korea; 3 Department of Industrial and Operations Engineering, University of Michigan, Ann Arbor, MI, United States of America; University of Florida, UNITED STATES

## Abstract

The prefrontal cortex (PFC) is involved in cognitive control of motor activities and timing of future intensions. This study investigated the cognitive control of balance recovery in response to unpredictable gait perturbations and the role of PFC subregions in learning by repetition. Bilateral dorsolateral (DLPFC), ventrolateral (VLPFC), frontopolar (FPFC) and orbitofrontal (OFC) cortex hemodynamic changes induced by unpredictable slips were analyzed as a function of successive trials in ten healthy young adults. Slips were induced by the acceleration of one belt as the participant walked on a split-belt treadmill. A portable functional near-infrared spectroscope monitored PFC activities quantified by oxyhemoglobin (ΔO_2_Hb) and deoxyhemoglobin (ΔHbR) during the consecutive trial phases: standing, walking, slip-recovery. During the first 3 trials, the average oxyhemoglobin (ΔO_2_Hb_avg_) in the DLPFC, VLPFC, FPFC, and OFC cortex was significantly higher during slip-recovery than unperturbed walking or the standing baseline. Then, ΔO_2_Hb_avg_ decreased progressively from trial-to-trial in the DLPFC, VLPFC, and FPFC, but increased and then remained constant in the OFC. The average deoxyhemoglobin (ΔHbR_avg_) presented mirror patterns. These changes after the third trial were paralleled by the progressive improvement of recovery revealed by kinematic variables. The results corroborate our previous hypothesis that only timing of the onset of a “good enough recovery motor program” is learned with practice. They also strongly support the assumption that the PFC contributes to the recall of pre-existing motor programs whose onset timing is adjusted by the OFC. Hence, learning is clearly divided into two steps delineated by the switch in activity of the OFC. Additionally, motor processes appear to share the working memory as well as decisional and predictive resources of the cognitive system.

## Introduction

Motor tasks are generally driven by cognitive attentional control. Multiple imaging techniques have investigated the role played by the prefrontal cortex (PFC) in motor tasks. Recently, the development of functional near-infrared spectroscopy (fNIRS) technology [1], based on the quantification of oxyhemoglobin (ΔO_2_Hb) and deoxyhemoglobin (ΔHbR) dynamics (hemodynamics), has also permitted to show that PFC activity, which varies over time to reflect flexibility and adaptation [2, 3], is associated with multiple functions. These functions include the performance of and attention to motor tasks [4–6] decision making [7], learning and motor skill acquisition [3, 8, 9] and working memory [see 2, 10 for review]. In addition, Ono et al. [9] posited that the PFC “plays a crucial role in holding the intention of future behavior until the right timing for execution based on the environmental feedback”.

PFC activity has been associated with the control and adjustment of walking [11–15], and balance recovery from unpredictable perturbation while standing [16]. Anterior PFC activity has been associated with “storing future action plans and their timely retrieval” [17]. These investigations, however, have focused on common motor tasks and have not considered the PFC’s role in responding to sudden unpredictable perturbations of ongoing movements. Some studies have even assumed that some of the PFC’s properties affect motor recovery in response to perturbation, at a level corresponding to the progression of a learning process.

Recovery from abrupt perturbations while walking is critical to avoid fall-induced injuries. Hence, understanding the organization of the protective mechanisms underlying the body’s recovery from unpredictable perturbations may help to develop effective wearable technology and specific exercise routines for improving balance to prevent injuries.

Previously we have shown that a) an adequate recovery from unpredictable trips can be learned within 8 repeated trials, but can be initiated in the first trial if a vibrotactile cue is provided 250 ms prior to the perturbation [18, 19]; and b) PFC subregions including the dorsolateral prefrontal cortex (DLPFC), ventrolateral prefrontal cortex (VLPFC), frontopolar prefrontal cortex (FPFC), and orbitofrontal cortex (OFC) are involved in the trip recovery process, and no significant increase in PFC activity is induced by vibrotactile cues (Lee et al. not published yet).

Building on our previous findings, in this study we investigated how the activity of PFC subregions evolves with the learning of slip recovery by trial repetition, and the association between these changes and the kinematic and timing parameters characterizing the improvement of the motor performance.

We used the repetition paradigm/block design to formulate three hypotheses. First, having hypothesized that during practice by repetition it is sufficient to learn the timing of initiation of a “generic good enough motor program” [19], we hypothesized that if this applies to slip recovery, the recovery step time response should decrease with slip trial repetition and thus improve kinematic behavior. Second, since PFC activity, as indicated by hemodynamic changes, decreases while learning a motor task [1, 20, 21] or after learning, we hypothesized that while learning the timing of the recovery response, PFC oxyhemoglobin concentration (ΔO_2_Hb) should decrease progressively from trial to trial, and the decreasing profile of ΔO_2_Hb should correlate or match with the progressive improvement of motor performance indicators. The opposite effects are expected from the deoxyhemoglobin (ΔHbR). Third, we hypothesized that changes in PFC activity may differ between the subregions and thus provide clues about the respective roles of the subregions.

## Materials and methods

### Participants

Ten healthy young adults (5 females and 5 males; age: 22.7 ± 3.2 yrs; stature: 174.9 ± 8.0 cm; weight: 69.1 ± 11.6 kg) participated in this study. All were naïve to the purpose of the experiment, and had never participated in studies of gait perturbations. Exclusion criteria included: self-reported neurological disorders (e.g., stroke, Parkinson’s disease, etc.); musculoskeletal dysfunctions; peripheral sensory diseases (e.g., peripheral neuropathy, Type 2 diabetes, etc.); pregnancy; left-footedness (kicking foot in response to a rolling ball); and a body mass index (BMI) greater than 30 kg/m^2^ because BMIs over 30 may affect gait [22–24]. The study protocol was approved by the University of Houston Institutional Review Boards, which accords with the Helsinki Declaration. Prior to the study, each participant reviewed and signed an informed consent form.

### Instrumentation

**Fig 1:**
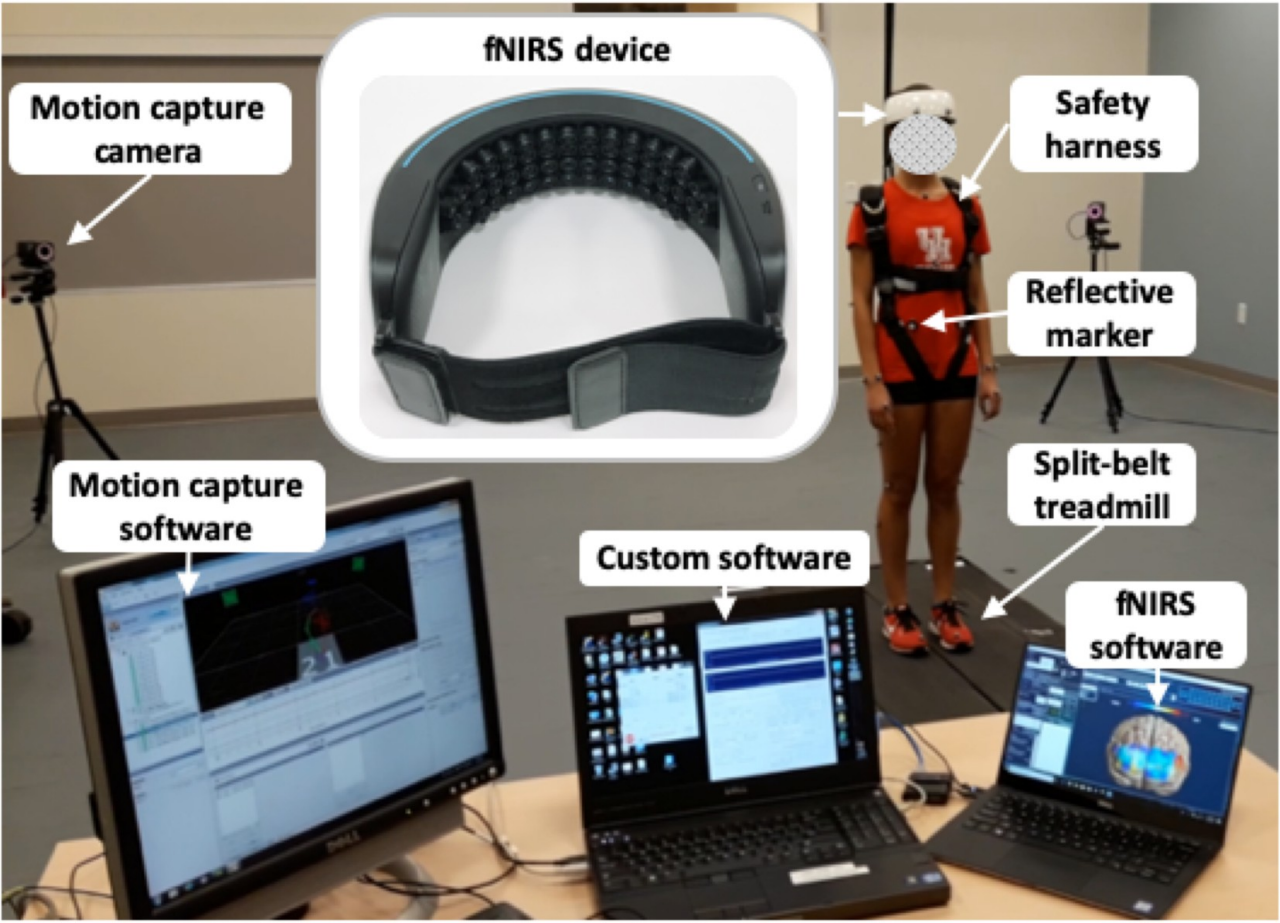
Experimental pieces of apparatus.

The equipment shown in Fig 1 has been used recently [25, 26]. The fall-inducing platform including a programmable split-belt treadmill equipped with two force plates located beneath each belt (Bertec, Columbus, OH, USA) and controlled by our custom software [18, 19, 27] was used to induce slips by accelerating the left belt in the anterior direction (i.e., the perturbation occurred at the foot level). A 48-channel wireless wearable fNIRS device (NIRSIT, OBELAB Inc., Seoul, S. Korea) placed on the forehead recorded the hemodynamic changes during each trial. This system is composed of laser and silicon photodiode with source—detector distances of 3 cm and operating at a scan rate of 8.138 Hz. A global signal regression is used to minimize systemic body oscillations. A 12-camera motion capture system (Vicon Motion Systems, Oxford, UK) recorded the displacement of 35 reflective markers commonly used to measure the body segment kinematics. Nexus 1.8 software was used to sample the positions of the markers and the ground reaction forces (GRFs) from the two force plates at a rate of 100 Hz. Our custom software generates start, stop and event signals to synchronize all data acquisitions, via a NI-6211card (National Instruments, Austin, TX, USA) for the motion capture system and the fNIRS device.

### Experimental protocol

All participants were outfitted with 35 reflective markers attached to body landmarks (head, neck, shoulders, arms, trunk, knees, and feet) to match the requirement of the Plug-in-Gait model [28] and wore the fNIRS device as well as an adjustable safety harness. Each participant self-selected a walking speed by adjusting the treadmill speed (average 0.9 ± 0.1 m/s). Before the trials, the fNIRS device was calibrated to prevent signal saturation from biological factors (e.g., skin and hair color, skin and skull thickness, etc.) [29].

All participants completed 6 consecutive trials in which the slip perturbation was induced randomly. Each trial included three periods: standing (15 s quiet standing); steady state walking at self-selected speed (the duration varied based on the randomness of the perturbation); and post-perturbation period (from the perturbation onset to the end of the trial time). The slip perturbation was applied randomly to the left foot but only during the loading phase (10% of the gait cycle corresponding to the initial double-limb support [19, 30] between the 31st and 40th step by accelerating the left belt in the anterior direction at a rate of 10 m/s^2^, to induce a backward slip. The accelerated belt returned to the pre-perturbation speed with the first heel strike of the right foot (i.e., the first response step of the non-slip foot), because stepping is the most common recovery response [31–33]. All trials ended 10 steps after the perturbation to account for the number of steps required to return to normal walking [19, 30] and the latency of the hemodynamic response subsequent to the perturbation (approximately 4 to 7 s) [34]. Normal walking resumed within 3 to 4 steps after a slip, corresponding to 4.2 ± 0.9 s. In all trials, during standing and walking, participants were required to fix their gaze on an “X” placed approximately 4.5 m ahead at eye level to stabilize their posture and minimize head movements and side-to-side walking variations [25, 26]. Consecutive trials were separated by a rest period (20 s) to allow brief relaxation of the torso and upper and lower extremities. Participants were given no information about the slip perturbation onset.

### Data processing

**Fig 2:**
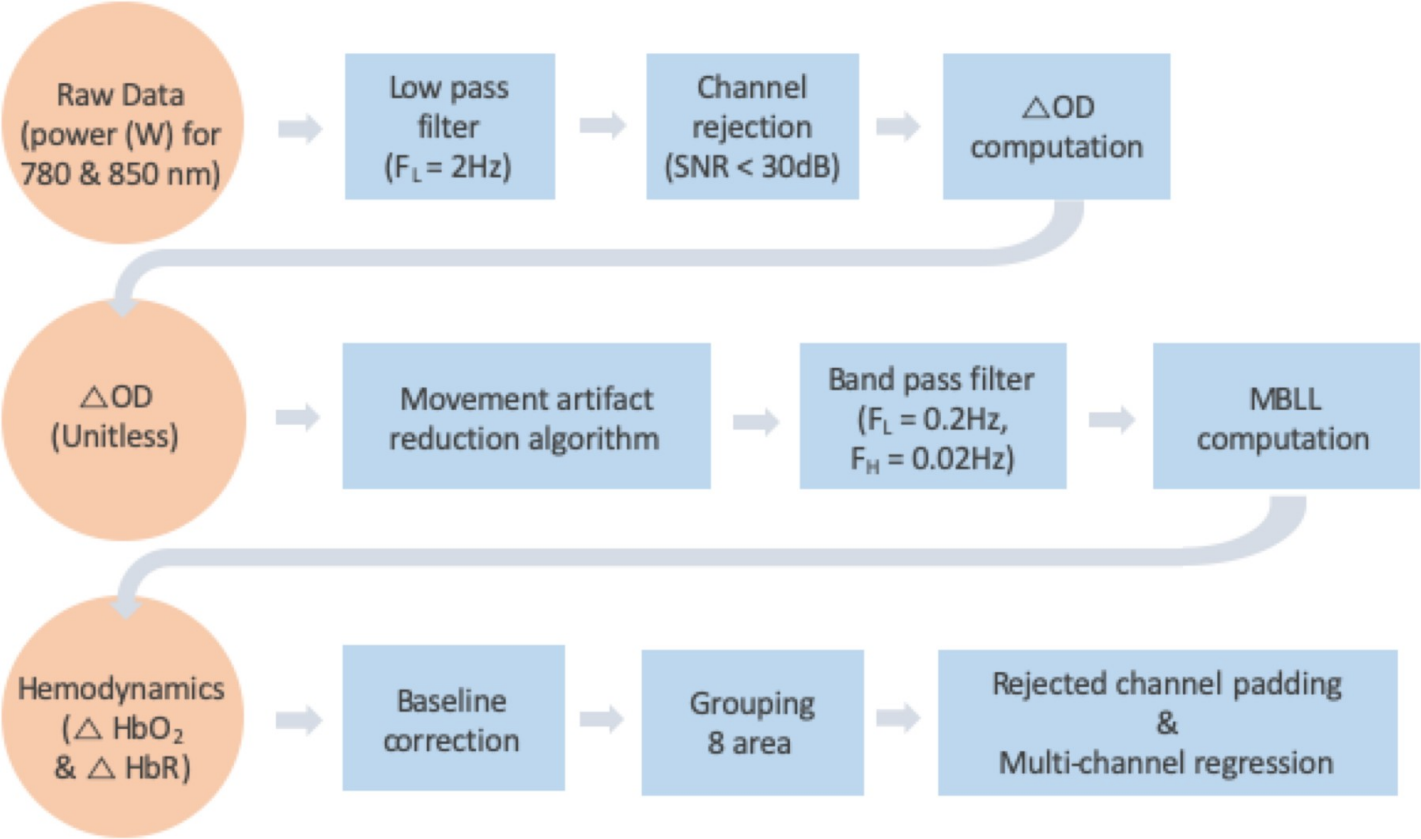
Flowchart of fNIRS signals processing. SNR = signal noise ratio; OD = optical density; MBLL = modified Beer-Lambert law.

**Fig 3:**
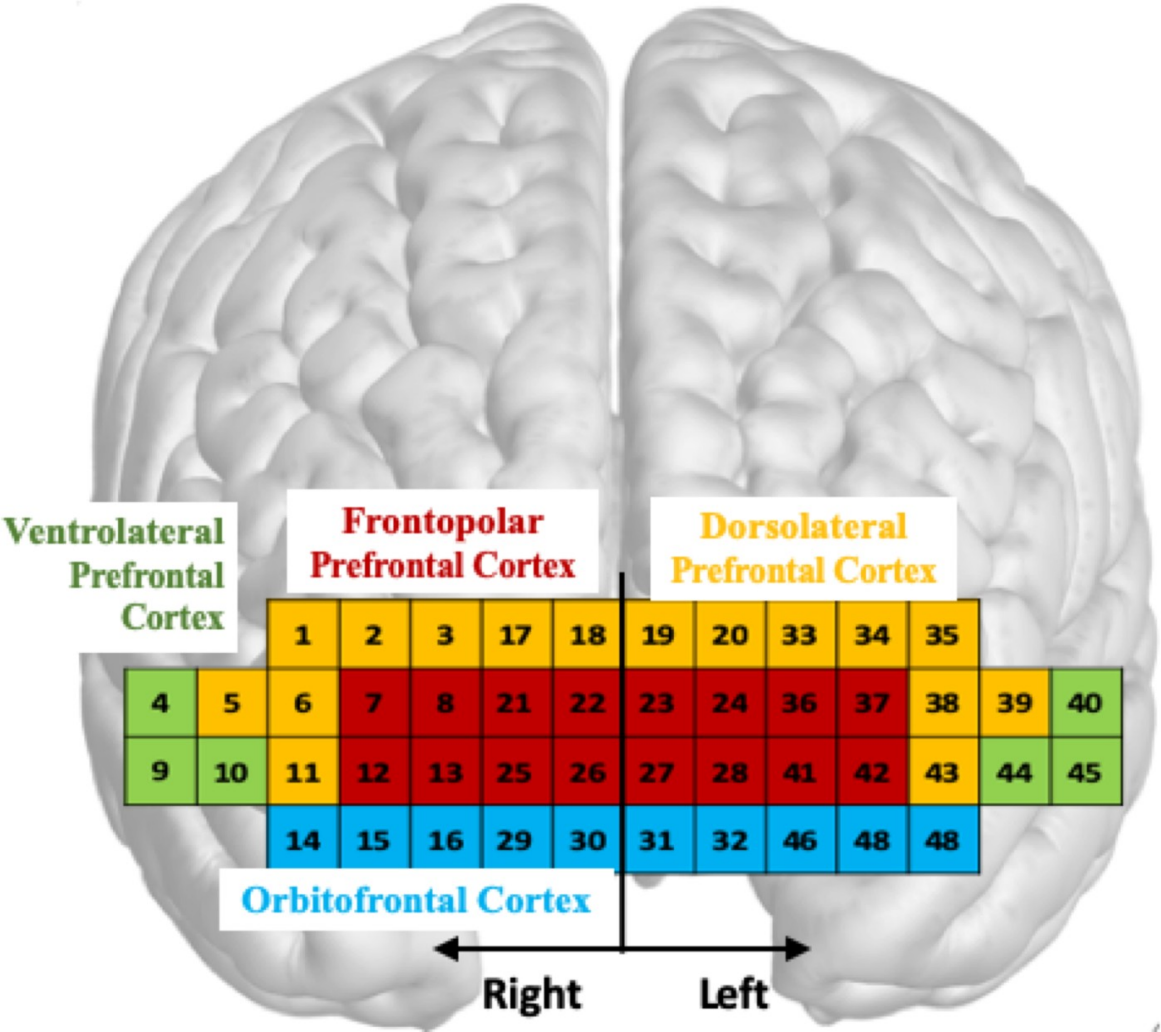
fNIRS channels. Array and subregions mapping: Dorsolateral PFC (yellow); Frontopolar PFC (red); Ventrolateral PFC (green); Orbitofrontal PFC (blue).

Whole body kinematics were computed by the Plug-in-Gait model in Nexus 1.8 and MATLAB (The MathWorks, Natick, MA, USA) was used to process the fNIRS data and GRFs. PFC activity was quantified by oxyhemoglobin (ΔO_2_Hb) and deoxyhemoglobin (ΔHbR) concentrations, since an increase in PFC activity is associated with an increase in ΔO_2_Hb and a concomitant decrease in ΔHbR [35]. The series of processing methods applied to both signals are shown in Fig 2. All 48 channels of the fNIRS’ raw data (i.e., received optical power of the used wavelength pair: 780 nm and 850 nm with emitting power of less than 1 mW) were low pass filtered at 2Hz. This cut-off frequency was derived from a power spectral density analysis. The threshold of a signal-to-noise ratio (SNR) of less than 30 dB was applied to reject unreliable or distorted channels caused by environmental noise; SNR was defined as the ratio of a mean to a standard deviation of the fNIRS’s filtered data for the first 5 s of the standing period (baseline period). The SNR threshold was set based on a previous finding that it corresponded to an effective extraction of reliable ΔO_2_Hb and ΔHbR signals in the presence of noise frequencies less than 0.1 Hz caused by blood circulation [36]. The average number of rejected channels in each trial was 5.0 ± 2.3. Next, the changes in optical density (ΔOD) relative to the baseline period were computed for the accepted channels [37]. Since fNIRS is sensitive to motion artifacts [38], reducing the effects on ΔOD caused by head movements during walking and slip perturbations was performed by the well validated movement artifact reduction algorithm (MARA) [39]. A threshold for the moving standard deviation time series of the MARA was set to 1 μM [39]. The computed ΔOD was filtered by a 0.02–0.2 Hz band pass filter to remove respiration, heart pulsation, equipment noise, and other irrelevant physiological effects [40]. The modified Beer-Lambert law (MBLL) was applied to compute ΔO_2_Hb and ΔHbR [37]. The differential path length factor (DPF) values of 780 nm and 850 nm are 5.075 and 4.64 in respectively. These values were referred from Choi et al. [41] that calculated DPF value by using the equations from Boas et al. [42]. The outcome of these processing steps is illustrated by an example presented in S1 Fig to illustrate the efficient filtering of the various artifacts before subsequent computation. Normalization (aka baseline correction) was performed by subtracting an average of ΔO_2_Hb and ΔHbR corresponding to the baseline period (i.e., the first 5 s of the standing period) from the computed ΔO_2_Hb and ΔHbR for each measuring period. The 48 channels were grouped into eight subregions: right and left DLPFC, VLPFC, FPFC, and OFC, as illustrated in Fig 3. For grouping, MNI transformation was conducted by individual location coordinates in 4 positions: nasion (Nz), right pre-auricular (RPA), left pre-auricular (LPA), and central zero (Cz) [43]. Rejected channels were padded by the average signal of the accepted channels within each subregion. A multi-channel regression method (global regression) was applied to each subregion to reduce possible signal contamination caused by extra-brain artifacts (e.g., scalp and systemic blood flow) [44]. Next, the average ΔO_2_Hb and ΔHbR (i.e., ΔO_2_Hb_AVG_ and ΔHbR_AVG_) within each subregion were computed for each period (standing, walking, recovery); for these measures, the time corresponding to the recovery period onset was shifted by a latency value computed as the time between perturbation onset and PFC activity onset. PFC activity onset was detected using the Moving Average Convergence Divergence (MACD) filter [45]. To simplify data analysis, only latency values for bilateral DLPFC, VLPFC, and FPFC were averaged for each trial, since OFC activity presents a specific pattern, as described in the results section.

**Fig 4:**
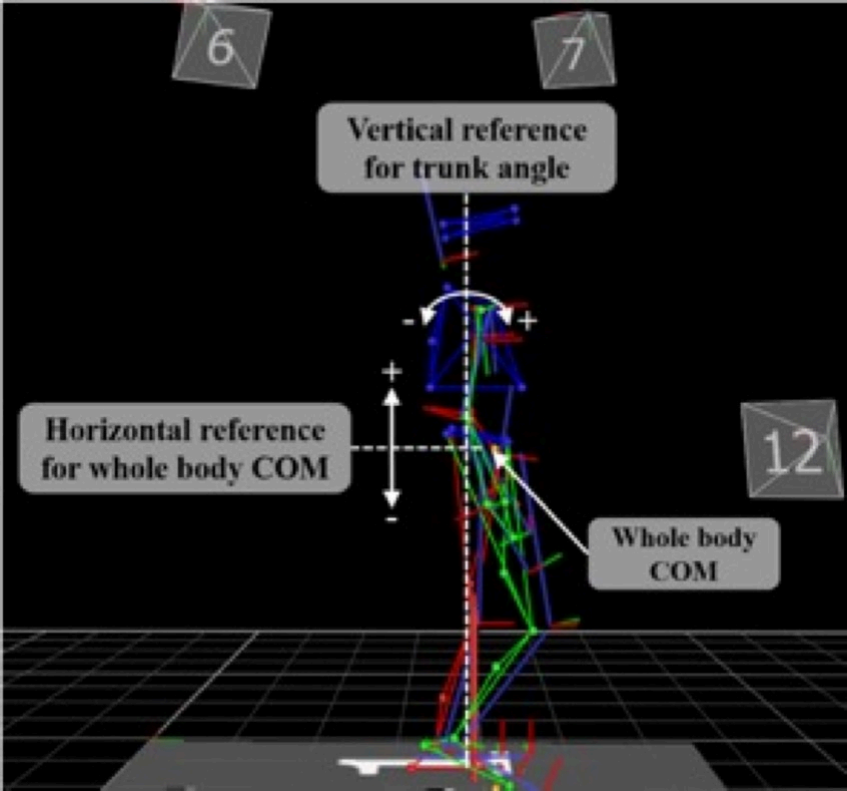
Reference lines for torso kinematics. Lines are superimposed on a representative body segments skeleton derived from the motion capture system.

The body kinematics and GRF signals were low pass filtered at 10 Hz [46]. To assess kinematic and kinetic responses following slip perturbations, trunk angular dispersion (AD), trunk range of motion (ROM), COM ROM, minimum COM (Min COM) position, and response step time of the right foot (i.e., non-slip foot) were quantified [19, 25–27]. Trunk AD, trunk ROM, COM ROM, and Min COM position were computed as an average during the standing, walking, and recovery periods [19]. References and planes in which the values are computed are shown in Fig 4. Trunk AD corresponds to one standard deviation of the trunk angular displacements in the sagittal plane. Trunk ROM quantifies the motion magnitude in degrees between the flexion and extension extrema in the sagittal plane. Since the participants differed in height, COM positions were normalized to the initial COM position in the sagittal plane. COM ROM indicates the magnitude of motion in cm between the maximum elevation and minimal depression along the vertical direction with respect to the horizontal reference. The response step time from the start of the slip perturbation to the first heel strike of the non-slip foot (i.e., right foot) is extracted from the GRFs [19, 30]. All variables were computed for each period, except latency and response step time that were computed only for the recovery period.

### Statistical analyses

Minitab^®^ (PennState University, PA, USA) was used to perform statistical analyses. The analysis was applied to oxyhemoglobin (ΔO_2_Hb) and deoxyhemoglobin (ΔHbR), as recommended recently [34, 47, 48], and to kinematic variables. Initially, a three-way ANOVA assessed the effects of gender, period (standing, walking, recovery) and trial (1–6) for all metrics. The Shapiro-Wilk test confirmed that the outcome measures were normally distributed. Gender was not a significant factor (*p* > 0.1) for any of the outcome measures, so one-way ANOVAs were applied to all variables to determine both the effect of period for the first trial and repeated measure ANOVAs were initially used to determine the effect of trial in the recovery period. Latency, and response time were analyzed as a function of trial only for the recovery period. Post hoc analysis using the Tukey-Kramer method (multiple comparisons) compared the differences between periods and between trials, respectively. Then, due to a conspicuous break point in all hemodynamic profiles as a function of trial in the recovery period, particularly for the OFC subregion, piecewise regressions (trial 1–3 and 3–6) were fitted to all outcome measures using trial 3 as the break point. Finally, the strength of association between averaged PFC activity (all regions) and averaged kinematic variables was measured by the Pearson’s correlation coefficient. Significance was set at *p* < 0.05.

## Results

### PFC hemodynamics

As expected, the concentrations in oxyhemoglobin (ΔO_2_Hb) and deoxyhemoglobin (ΔHbR) varied concomitantly in opposite directions. For simplicity, as the results were identical in term of significance, only ΔO_2_Hb_AVG_ variations are presented. Results corresponding to ΔHbR_AVG_ are illustrated in supporting documents. The respective ANOVAs applied to ΔO_2_Hb_AVG_ indicated a significant effect of the period for the first trial (Table 1A) and a significant effect of the trial for the recovery period (Table 1B) for all PFC subregions with the exception of OFC. Identical outcomes were obtained for ΔHbR_AVG_.

**Table 1:** ANOVAs applied to oxyhemoglobin data.

PFC subregions	Effect	DF	F	Pr > F	Partial Eta Squared
**A.** ANOVA applied to ΔO_2_Hb_AVG_ for Periods, 1^st^ trial					
**DLPFC**_**L**_	Period (3)	2, 9	26.127	.000*	0.659
**DLPFC**_**R**_	Period (3)	2, 9	26.567	.000*	0.663
**VLPFC**_**L**_	Period (3)	2, 9	24.848	.000*	0.648
**VLPFC**_**R**_	Period (3)	2, 9	27.090	.000*	0.667
**FPFC**_**L**_	Period (3)	2, 9	43.073	.000*	0.761
**FPFC**_**R**_	Period (3)	2, 9	48.141	.000*	0.781
**OFC**_**L**_	Period (3)	2, 9	.720	.496	0.051
**OFC**_**R**_	Period (3)	2, 9	.346	.710	0.025
**B.** ANOVA applied to ΔO_2_Hb_AVG_ for recovery period, all trials					
**DLPFC**_**L**_	Trial (6)	2, 59	4.145	.003*	0.277
**DLPFC**_**R**_	Trial (6)	2, 59	3.326	.011*	0.235
**VLPFC**_**L**_	Trial (6)	2, 59	4.207	.003*	0.280
**VLPFC**_**R**_	Trial (6)	2, 59	3.891	.004*	0.265
**FPFC**_**L**_	Trial (6)	2, 59	3.956	.004*	0.268
**FPFC**_**R**_	Trial (6)	2, 59	4.593	.001*	0.298
**OFC**_**L**_	Trial (6)	2, 59	10.634	.000*	0.496
**OFC**_**R**_	Trial (6)	2, 59	7.524	.000*	0.411

PFC subregion sides are identified by _**L**_ = left, _**R**_ = right. α = 0.05,

* = significance.

**Fig 5:**
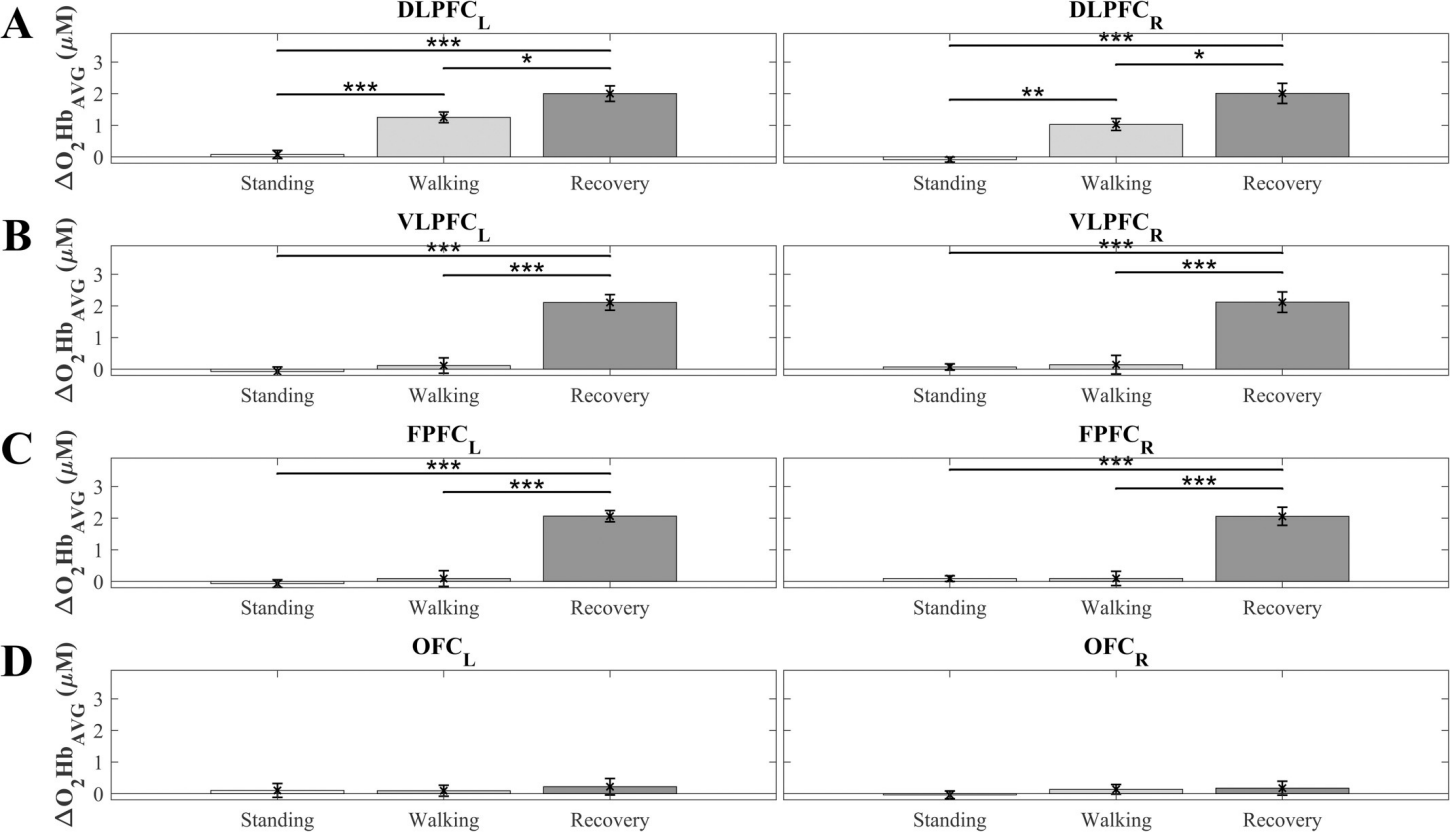
Oxyhemoglobin concentrations (ΔO_2_Hb_AVG_) for the first slip trial. Average values across all participants (N = 10) for left and right prefrontal cortex (PFC) subregions (top to bottom panels) as a function of the period (standing, walking, recovery): A) Left and right dorsolateral PFC (DLPFC_L/R_); B) Left and right ventrolateral PFC (VLPFC_L/R_); C) Left and right frontopolar FPFC (FPFC_L/R_); D) Left and right orbitofrontal cortex (OFC_L/R_). Error bars indicate standard error of the corresponding means. Asterisks: significant differences (* p < 0.05, ** p < 0.01, *** p < 0.0001).

**Fig 6:**
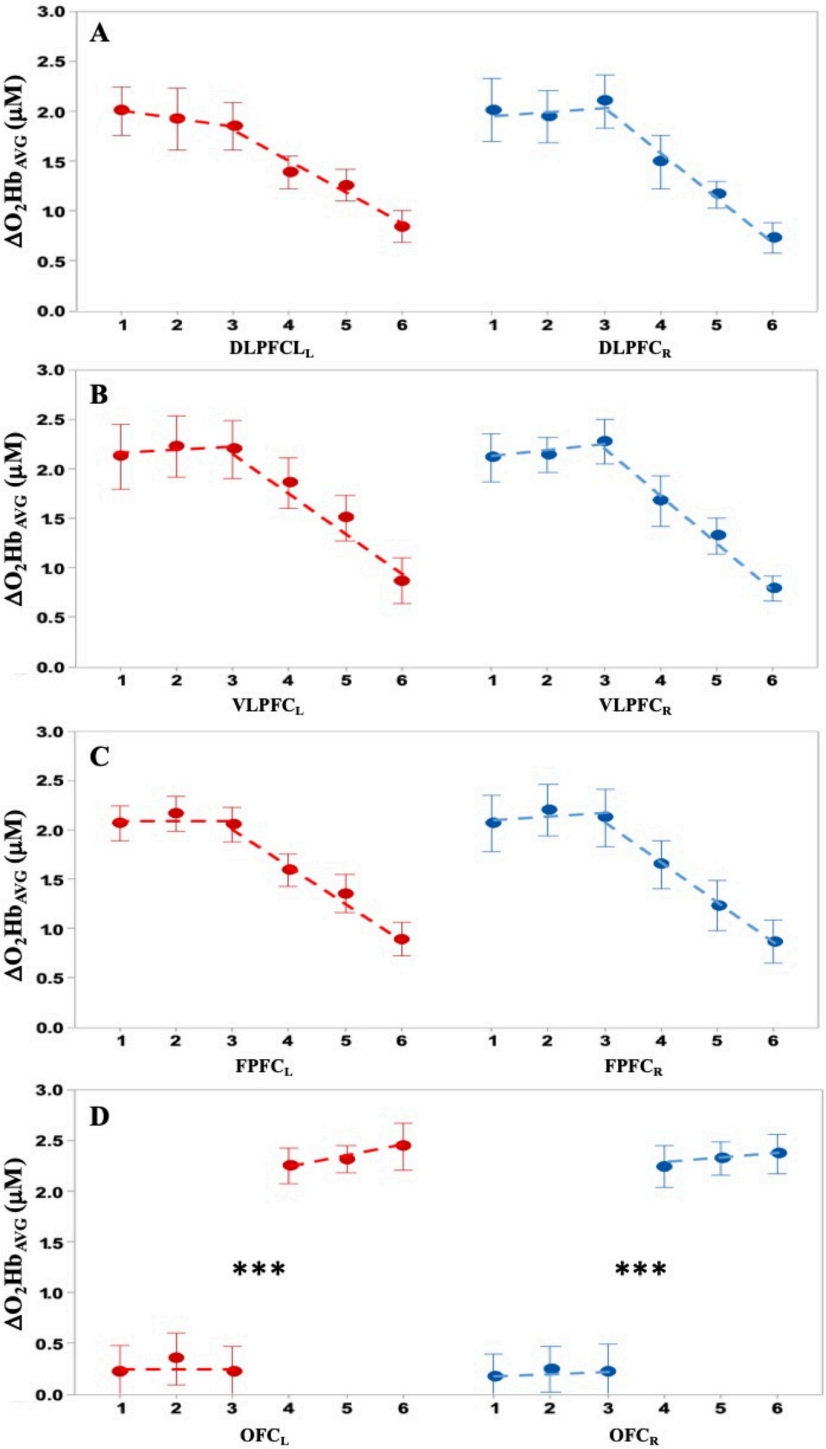
Oxyhemoglobin concentrations (ΔO_2_Hb_AVG_) as a function of trials. Average values across all participants (N = 10) for left (●) and right (●) prefrontal cortex (PFC) subregions (top to bottom panels) as a function of the trial number: A) Left and right dorsolateral PFC (DLPFC_L/R_); B) Left and right ventrolateral PFC (VLPFC_L/R_); C) Left and right frontopolar PFC (FPFC_L/R_); D) Left and right orbitofrontal cortex (OFC_L/R_). Error bars correspond to the standard error of the corresponding means. Piecewise linear regressions (---) use trial 3 as the break point (see bottom graph and text for justification). *** p < 0.0001.

A representation of hemodynamic changes and significant differences as a function of the period for the first slip trial is shown in Fig 5. Activity in the whole PFC/any subregion was negligible while standing. In the DLFPC, the activity increased significantly (*p* < 0.01) bilaterally during walking and increased significantly (*P* < 0.05) again during recovery. Activity in the VLPFC and FPFC increased only during recovery (*p* < 0.00001); very small increases in activity in the OFC as a function of period were insignificant. ΔHbR_AVG_ presented a mirror effect (see S2 Fig). The patterns of changes in hemodynamics and significant differences as a function of trials for each PFC subregion of the recovery period are shown in Fig 6. There was a negligible non-significant (*p* >0.1) difference between the first three trials in all PFC subregions followed by a monotonous decrease in ΔO_2_Hb_AVG_ as a function of trial for the DLFPC, VLFPC, and FPFC (piecewise regressions), while concomitant activity in the OFC switched significantly (*p* < 0.000) from a low level to a higher level and remained at the same level over the last 3 trials (Fig 6). This feature is supported by the piecewise linear regressions, with higher r^2^ compared to corresponding single linear regressions (Table 2A). ΔHbR_AVG_ showed a mirror effect (see S3 Fig).

**Table 2:** Comparisons of piecewise and single linear regressions.

**A**	**R**^**2**^	
**PFC subregions**	Piecewise (T_3-6_)	(T_1-6_)
DLPFC_L_	0.99	0.93
DLPFC_R_	0.98	0.83
VLPFC_L_	0.97	0.78
VLPFC_R_	0.87	0.80
FPFC_L_	0.98	0.87
FPFC_R_	0.99	0.84
**B**	**R**^**2**^	
**Kinematics**	Piecewise (T_3-6_)	(T_1-6_)
TAD	0.97	0.82
TROM	0.97	0.84
COM ROM	0.97	0.88
Min COM	0.96	0.85
RST	0.98	0.92

T_x-y_ = Trials included in regression.

TAD = Trunk angular dispersion, TROM = trunk range of motion, COMROM = center of mass range of motion, MIN COM = Minimum of COM, RST = response step time.

### Kinematics

**Fig 7:**
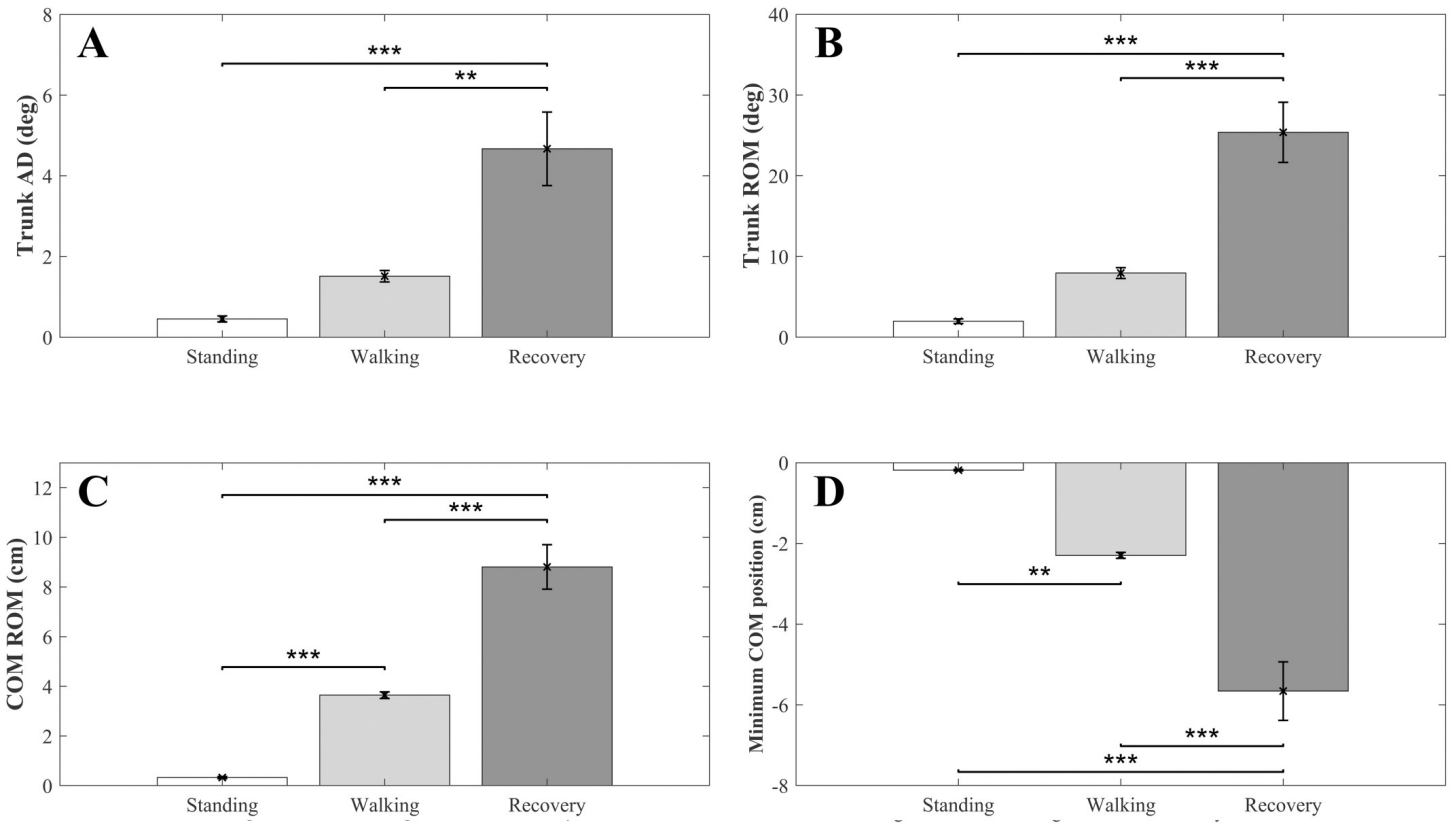
Kinematic variations for the first trial. Average values across all participants (N = 10) as a function of period: A) Trunk angular dispersion (TAD); B) trunk range of motion (TROM); C) center of mass range of motion (COM ROM); D) Minimum of COM (Min COM). Error bars indicate standard error of the corresponding means. Asterisks: significant differences (* p < 0.05, ** p < 0.01, *** p < 0.0001).

The changes in kinematics (TAD, TROM, COM ROM, Min COM, and right foot RST) as a function of period in the first trial are shown in Fig 7. As expected, the magnitude of each variable was significantly greater in the recovery period than in the walking (*p* < 0.001) and standing (*p* < 0.0001) periods. COM ROM and Min COM magnitudes were also significantly greater (*p* < 0.001) in the walking than in the standing period. The increase in trunk sway and ROM magnitudes in healthy young adults was insignificant between standing and walking at the self-selected slow speed because stability was not compromised during this walking condition.

**Fig 8:**
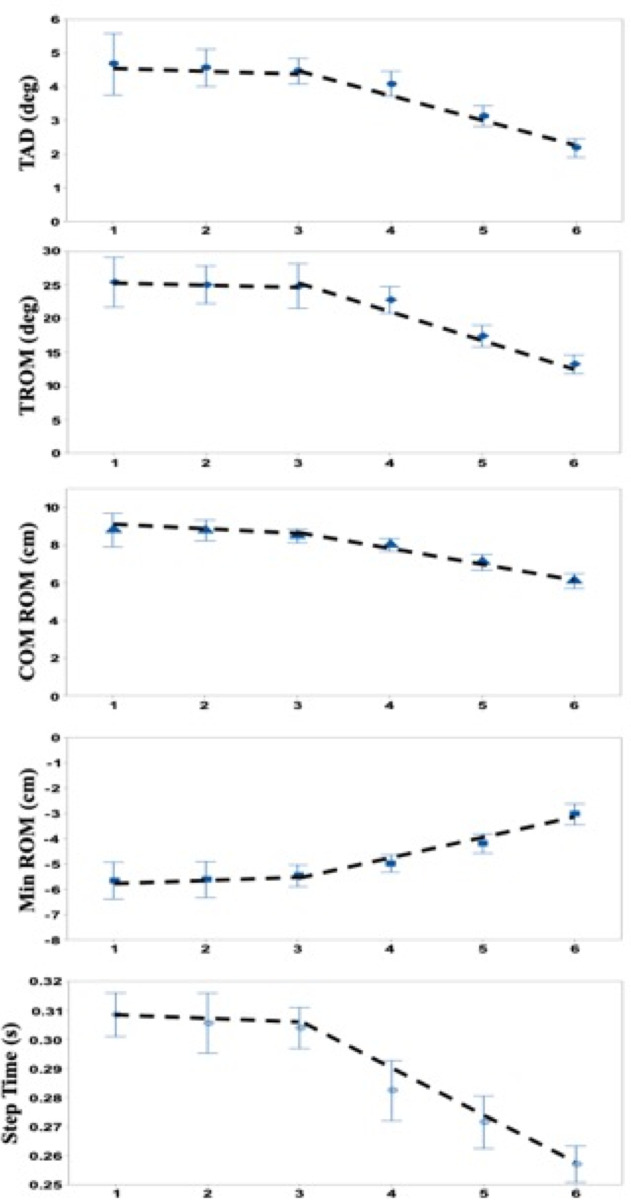
Kinematic variations and response time as a function of trials. Average values across all participants (N = 10). From top to bottom: TAD = Trunk angular dispersion; TROM = Trunk range of motion; COM ROM = Center of mass range of motion; Min ROM = Minimum of COM; Step Time = first response step time. Error bars correspond to the standard error of the corresponding means. Piecewise linear regressions (**---**) use trial 3 as the break point (see Fig 5 and text for justification).

Changes in kinematics variables and response step time as a function of trial in the recovery period are shown in Fig 8. As observed for PFC activity (Fig 6), the value of all variables (OFC excepted) associated with recovery movements were insignificant for the first 3 trials (*p* > 0.1) and then evolved progressively trial after trial in the direction of recovery improvement/stability (Fig 8). These change pattern similarities are supported by the piecewise regression analysis, with higher r^2^ compared to corresponding single linear regressions (Table 2B).

### PFC hemodynamics-kinematics correlations

Correlations between average hemodynamics and kinematics measurements, including response step time, were computed to further demonstrate the association between cognitive learning and motor processes. Details of individual subregions correlations with kinematic variables are presented in S1 and S2 Tables. For simplicity here, since the pattern of changes in DLPFC, VLPFC, FPFC with learning (trial #) were symmetric (left vs right) and similar (Fig 6) the activities of these subregions were combined into an overall average to represent the global activity. The OFC activity was not included as the role of this area, which will be discussed specifically, appears to be very distinct. The computation showed that all measures related to kinematics correlated significantly with PFC (DL,VL,F) activity (|correlation coefficient| > 0.98, *p* < 0.0001) as shown in Table 3. Due to the inherent sign of Min COM relative to the standing reference level, the correlation was negative for that variable. Correlations ≥ |0.80| were considered as high. Furthermore, the invariance before and after the flip in the OFC activity (Fig 6) indicates that spurious hemodynamic activity that could have been associated with motion induced physiological effects are not of significant importance. This allows to rule out their eventual influence on correlations and emphasize a parallelism between cognitive processes and motor responses.

**Table 3:** PFC (DL,VL,F)—kinematics correlations.

	TAD	TROM	COMROM	Min COM	RST
PFC (DL,VL,F)	0.980	0.981	0.981	-0.983	0.991
	0.000	0.000	0.000	0.000	0.000

Cell Contents

Pearson correlation

P-Value

TAD = Trunk angular dispersion, TROM = trunk range of motion, COMROM = center of mass range of motion, Min COM = Minimum of COM, RST = response step time.

## Discussion

Differences between periods (standing, walking, recovery) and successive trials (1–6) were significant for all outcome measures. Parallel changes in kinematic, kinetic, and hemodynamic data in the recovery period, trial after trial, show an association between PFC activity and motor behaviors while learning. However, learning appears to be effective only after a number of “*observations*”. Furthermore, specific significant differences in hemodynamics between the different PFC subregions can be associated with different functions.

The contributions of the PFC subregions to slip recovery and learning an adapted strategy are clearly differentiated (Figs 5 and 6). Initially, when compared to the standing reference, for which PFC activity (ΔO_2_Hb_avg_) is negligible, DLPFC activation is significantly while walking and even greater during slip recovery. The VLPFC and FPFC, however, are highly activated only during the recovery, while OFC activity does not appear to increase much during walking or slip recovery. During learning by trial repetition, which, according to all kinematic and hemodynamic indicators, is evident only after the third trial, the VLPFC and FPFC activity decreases progressively while the OFC activity switches to a significantly higher level, and then remains invariant. Hence, when unpredictable movement perturbations occur, a two-stage decision process appears necessary.

One distinction between the DLPFC and the other subregions is that the DLPFC is largely implicated in walking whereas the VLPFC, FPFC, and even the OFC are involved only minimally (Fig 5). The absence of a significant increase in activity in these latter subregions when walking (compared to standing) is not surprising since walking is largely automatized (see discussion below on redistribution of activities with learning) and requires little cognitive control [8], at least in healthy young adults; older adults or individuals affected by disorders may need more cognitive control [34, 49]. In contrast, the increase in activity when walking indicates that despite automatization, walking requires some attentional demand associated with maintaining both the walking task goal and the active dynamic balance control [see 50 for review], and in the present context, the likely expectation of a perturbation. As cognitive control may need to be higher for treadmill than over the ground walking [51] it is logical to assume that within a similar context, the observed PFC activities could slightly differ in magnitude but not in pattern in a ground walking context. The role of monitoring appears to be played by the DLPFC alone without burdening the other subregions. Similarly, the results obtained by Koenraadt et al. [13], which were found for a comparable walking period of 35 seconds, but for the whole PFC, show significant PFC activity during the first part of the walking period and a decrease in activity during the last part of the walking period. This difference supports our assumption that a certain level of attention needs to be maintained as long as a perturbation, although theoretically unpredictable, is anticipated. From these patterns of PFC activity two types of differentiations can be distinguished: between phases (pre-learning vs learning) and between subregions as discussed below.

### Pre-learning phase

During the first three trials, the absence of changes in PFC activity (Fig 6) indicates an absence/no appearance of cognitive changes in response to the perturbation. This is corroborated by the absence of significant changes in strategy/ motor response dynamics and onset delay of recovery response (Fig 8). It is logical to assume that the correlated kinematic behaviors (Table 3) are the consequences of and not the cause of cognitive control. Hence, this “absence of initiative” phase may suggest that the first three trials may correspond to a necessary number of observations to develop/predict an adapted recovery strategy. This hypothesis is in agreement with the results of previous studies showing that movement learning under unpredictable mechanical perturbation [52, 53] or even cyclic repetition [54] is based on information from a few preceding trials that may improve performance. Clearly, using a few observations before initiating a predicted response is more computationally and cognitively efficient than initiating a succession of motor programs requiring multiple/substantial reorganization because the switching cost is not negligible from the motor [55, 56] and cognitive point of view [57]. Under this assumption, the absence of activity change in all PFC subregions is not surprising. Furthermore, recent studies also indicate that buffering of information is unlikely to occur in the PFC [see 10 for review], despite its acknowledged large role in working memory [10, 58–61], motor planning [62], task switching [63] and memory updating [64]. However, it cannot be fully dismissed that the lack of significance in changes of PFC activity could partly result from the limited resolution of the fNIRS and the inherent delays of responses. Although numerous studies have investigated motor learning and the role of the PFC in motor learning (as cited above), little attention has been paid to the first few trials. They are usually included in blocks (averages), although movement performance in the first block/batch is usually distinct from successive blocks [e.g., 54], it is still used to model learning as a continuous progressive process. Most learning models use an exponential fitting including the set of initial movements, regardless of the nature of the experiment (with or without unpredictable perturbations). According to our present and previous results [19], stepwise regressions would recognize the two steps in learning/adapting to unpredictable perturbation by acknowledging a discontinuity/non linearity in the process. In the case of unpredictable perturbations, we hypothesize that the pre-learning period of observation may be used to determine “what” must be adapted to improve the motor response, which is compatible with the demonstration that prediction based on an internal model [see 65 for example] precedes control in motor learning [66]. We also assume that “what” is not the recovery motor program itself, which is presumably accomplished by the DLPFC [67], but only the time at which a “good enough” recovery motor program must be triggered to rapidly regain stability [19]. Our assumption is strongly supported by the immediate adjustment of the recovery motor program when a “warning” cue is provided 250 ms before the mechanical perturbation [18].

### Learning phase

The improvement of performance with trials is associated with the switch of OFC activation and the parallel decrease in the DLPFC, VLPFC, and FPFC activity. The OFC has been associated with multiple roles including being a center of cognitive maps [68], “economic” decision making, based on a comparison of subjective values [69, 70], motivational, emotional and social behaviors [71], as well as being the “oracle” predicting behavioral outcomes [72] and learning that controls decision making [7, 73]. The OFC has also been claimed to regulate “online” goal-directed action selection based on the value of a reward [74]. Although these functions (decision, prediction, regulation) have been established from experiments exploring cognitive processes, the corresponding conclusions/assumptions may apply to the sensory-motor processes involved in movement planning, control and learning and not solely to sensory-cognitive or cognito-cognitive processes. Hence, as OFC activity switches only after the 3^rd^ trial and remains relatively constant thereafter, it is hypothesized that:

1. OFC activity, which is modulated and/or gated by different pathways [2, 75–77], is likely to occur subsequent to a disinhibition from a primary/higher decision center that delays its activation until the most efficient/appropriate program parameter to be adjusted by learning has been identified. This primary decision process may take place in the PFC, which holds intension for future action [9] and more particularly the DLFPC, which has a direct connection with the OFC [78] and contributes to the inhibition of inappropriate responses [79]. Note that the connectivity between structures and PFC subregions and its complexity is beyond the scope of the present study. Obviously, this process relies on the existence of at least a generic motor program/internal model [18] in the long term memory. This constitutes the prior knowledge to predict behavior [73]. This “good enough” recovery program is transferred to the working memory (PFC) and the OFC controls the adjustment of the onset delay.
2. OFC functions (i.e., estimation/prediction of the timing of the recovery motor program, prediction of outcome, reward associated with improvement of the motor outcome, decision to reduce timing delay) need to be performed at each iteration. This necessary cognitive control may explain why in this recovery phase the OFC activity remains similar over the subsequent trials, while activity decreases in the other subregions.

The decrease in DLPFC, VLPFC, and FPFC activity with learning (Fig 6) concurs with previous results obtained from diverse cognitive and motor tasks [1, 9, 80–82]. The decrease has been associated with a relaxation of cognitive control over motor performance during learning [83], and the correlated transfer of activity to other brain regions [3, 8, 80] and/or brain network reconfiguration to “facilitate different facets of cognitive computation” [84].

## Conclusion

The singularity of OFC activity relative to other PFC subregions led us to posit that learning includes two steps, at least in the context of unpredictable perturbations, and that processing requirements rather than the type of information (cognitive vs motor) to be processed produce the patterns of activity. Our hypothesis is in line with the understanding that before becoming more automatic (by learning), cognitive decisions must be made to “improve” their outcomes and that both cognitive (e.g., emotion, decision) and motor (motion) information share the PFC processors to a large extent. Reconfiguration of the network underlying the processing of information is associated with organizing the flow of information as a function of required processing [84]. The question remains though: what brain area orchestrates this plasticity? Or is reconfiguration subject to rules derived from life experience (learning) and set by the context and functionality of the elements of the network as proposed by Khambhati et al. [84]. This latter proposition is supported by a model based on machine learning and association of nodes as a function of their functionality. Here, the OFC appears as a necessary element of the network for the cognitive control of the timing of recovery motor programs.

## Limitations

Although providing consistent results positively supporting our hypotheses, the current study was performed with 10 participants, which does not represent a large cohort. The mechanisms underlying recovery from unpredictable slip (or trip) perturbation while walking focus on a concept applied to healthy young adults. It may be assumed that the corresponding generic process would be to some extent similar in healthy older adults. However, older adults may be affected by cognitive impairments that could influence the organization of their responses. Hence, investigations including such a group, as well as high risk of falling populations are necessary to further understand the implicated cognitive mechanisms. A study involving specific older patients is currently underway in our laboratory. The investigation was limited to a cognitive decisional aspect most likely taking place in the PFC. Hence, the roles/influences of other brain areas also involved in motor activities and learning [85] and more particularly in walking [49] are considered but not investigated in the present study. Finally, the use of a treadmill allowed to control the perturbation and results may slightly differ in over the ground walking; however, it would be expected that the concept and role of OFC remain the same.

## Supporting information

S1 Fig Time variations of ΔOD, ΔO_2_Hb, and ΔHbR. These variations are illustrated with and without the application of motion artifact removal algorithm (MARA). They correspond to a typical example obtained from one channel for one participant in one trial. **A**) Change in optical density (ΔOD) for the 780 and 850 nm wavelength pair during the walking and recovery periods. **B**) Computed corresponding Oxyhemoglobin (ΔO_2_Hb) and deoxyhemoglobin (ΔHbR) concentrations.
(DOCX)

S2 Fig Deoxyhemoglobin concentrations (ΔHbR_AVG_) for the first slip trial. Average values across all participants (N = 10) for left and right prefrontal cortex (PFC_L/R_) subregions (top to bottom panels) as a function of the period (standing, walking, recovery): **A**) Left and right dorsolateral PFC (DLPFC_L/R_); **B**) Left and right ventrolateral PFC (VLPFC_L/R_); **C**) Left and right frontopolar PFC (FPFC_L/R_); **D**) Left and right orbitofrontal cortex (OFC_L/R_). Error bars indicate standard error of the corresponding means. Asterisks: significant differences (* p < 0.05, ** p < 0.01, *** p < 0.0001).
(TIFF)

S3 FigDeoxyhemoglobin concentrations (ΔHbR_AVG_) as a function of trials.Average values across all participants (N = 10) for left (●) and right (●) prefrontal cortex (PFC) subregions (top to bottom panels) as a function of the trial number: **A**) Left and right dorsolateral PFC (DLPFC_L/R_); **B**) Left and right ventrolateral PFC (VLPFC_L/R_); **C**) Left and right frontopolar PFC (FPFC_L/R_); **D**) Left and right orbitofrontal cortex (OFC_L/R_). Error bars correspond to the standard error of the corresponding means. Piecewise linear regressions (---) use trial 3 as the break point (see bottom graph and text for justification). *** p < 0.0001.(DOCX)

S1 Table ΔO_2_Hb_AVG_ PFC subregions–kinematics correlations. *Cell Contents*. *Pearson correlation*. *P-Value*. TAD = Trunk angular dispersion, TROM = trunk range of motion, COMROM = center of mass range of motion, Min COM = Minimum of COM, RST = response step time.
(DOCX)

S2 Table ΔHbR_AVG_ PFC subregions–kinematics correlations. *Cell Contents*. *Pearson correlation*. *P-Value*. TAD = Trunk angular dispersion, TROM = trunk range of motion, COMROM = center of mass range of motion, Min COM = Minimum of COM, RST = response step time.
(DOCX)
