# Supplementary figures and images for: Roles of the prefrontal cortex in learning to time the onset of pre-existing motor programs
Source: PLoS One. 2020 Nov 9;15(11):e0241562. doi: 10.1371/journal.pone.0241562 (PMC7652266; doi:10.1371/journal.pone.0241562)

S1 Fig.


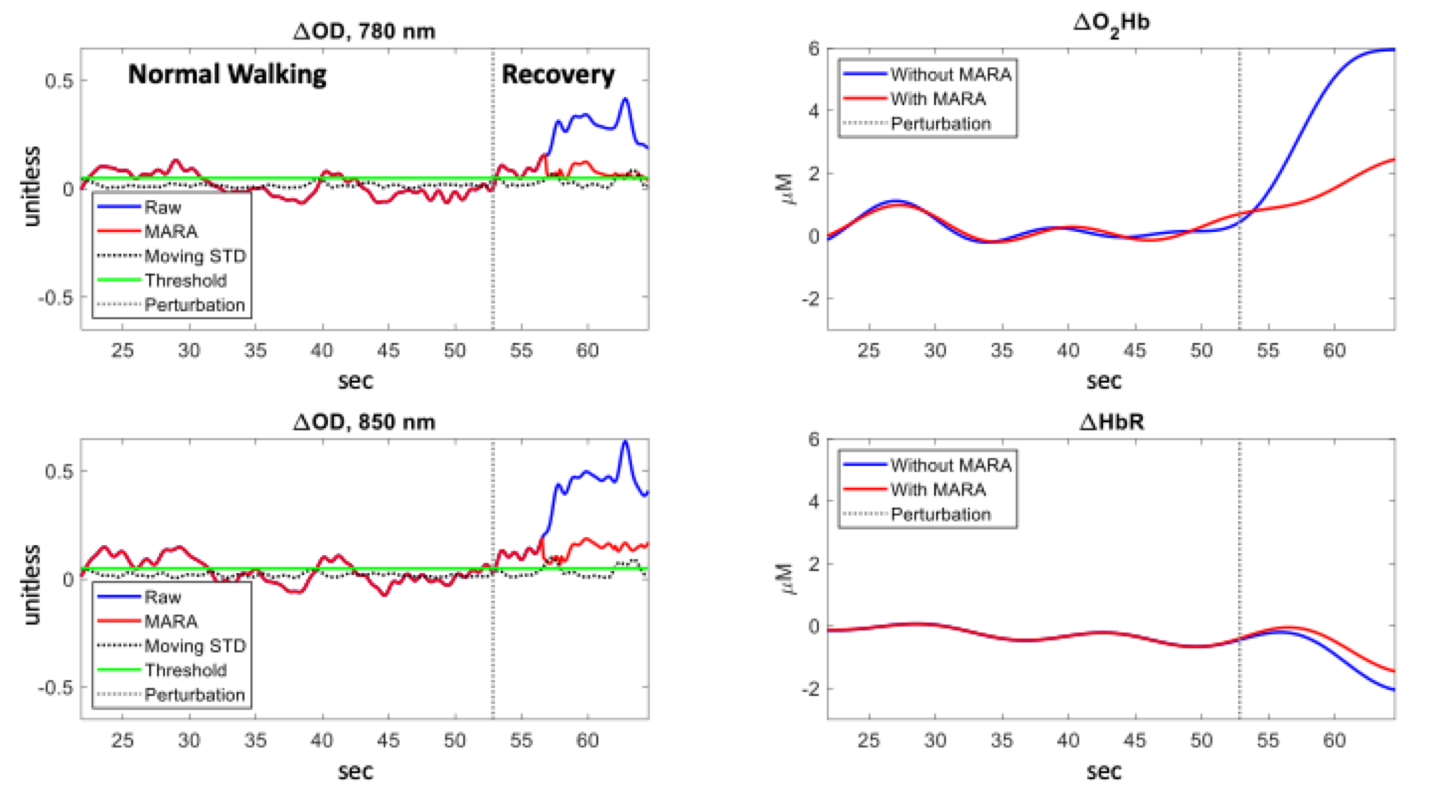

Supplement: S1 Fig — These variations are illustrated with and without the application of motion artifact removal algorithm (MARA). They correspond to a typical example obtained from one channel for one participant in one trial. A) Change in optical density (ΔOD) for the 780 and 850 nm wavelength pair during the walking and recovery periods. B) Computed corresponding Oxyhemoglobin (ΔO2Hb) and deoxyhemoglobin (ΔHbR) concentrations. (DOCX) [file pone.0241562.s001.docx]

S3 Fig


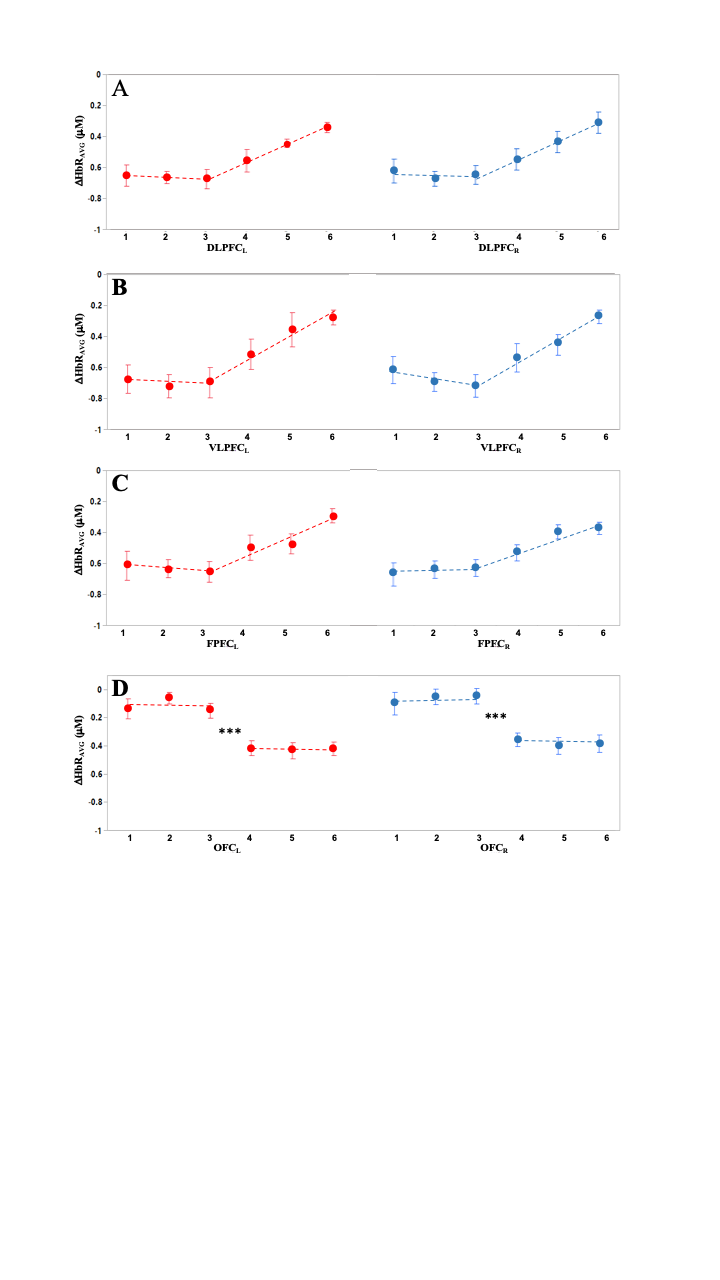

Supplement: S3 Fig — Average values across all participants (N = 10) for left (●) and right (●) prefrontal cortex (PFC) subregions (top to bottom panels) as a function of the trial number: A) Left and right dorsolateral PFC (DLPFCL/R); B) Left and right ventrolateral PFC (VLPFCL/R); C) Left and right frontopolar PFC (FPFCL/R); D) Left and right orbitofrontal cortex (OFCL/R). Error bars correspond to the standard error of the corresponding means. Piecewise linear regressions (---) use trial 3 as the break point (see bottom graph and text for justification). *** p < 0.0001. (DOCX) [file pone.0241562.s003.docx]
