# Supplementary material for: Roles of the prefrontal cortex in learning to time the onset of pre-existing motor programs
Source: PLoS One. 2020 Nov 9;15(11):e0241562. doi: 10.1371/journal.pone.0241562 (PMC7652266; doi:10.1371/journal.pone.0241562)
Supplement: S1 Table — Cell Contents. Pearson correlation. P-Value. TAD = Trunk angular dispersion, TROM = trunk range of motion, COMROM = center of mass range of motion, Min COM = Minimum of COM, RST = response step time. (DOCX) [file pone.0241562.s004.docx]

S1 Table. ΔO_2_Hb_AVG_ PFC subregions – Kinematics Correlations.

|  |  |  | **PFC subregions** | | |  |
| --- | --- | --- | --- | --- | --- | --- |
| Kinematic  Variables | DLPFCL | DLPFCR | VLPFCL | VLPFCR | FPFCL | FPFCR |
| TAD | 0.959 | 0.971 | 0.988 | 0.976 | 0.974 | 0.976 |
|  | 0.003 | 0.001 | 0 | 0.001 | 0.001 | 0.001 |
|  |  |  |  |  |  |  |
| TROM | 0.954 | 0.974 | 0.986 | 0.978 | 0.972 | 0.979 |
|  | 0.003 | 0.001 | 0 | 0.001 | 0.001 | 0.001 |
|  |  |  |  |  |  |  |
| COMROM | 0.971 | 0.972 | 0.986 | 0.975 | 0.983 | 0.983 |
|  | 0.001 | 0.001 | 0 | 0.001 | 0 | 0 |
|  |  |  |  |  |  |  |
| Min COM | -0.967 | -0.972 | -0.994 | -0.979 | -0.981 | -0.976 |
|  | 0.002 | 0.001 | 0 | 0.001 | 0.001 | 0.001 |
|  |  |  |  |  |  |  |
| RST | 0.995 | 0.989 | 0.96 | 0.98 | 0.99 | 0.987 |
|  | 0 | 0 | 0.001 | 0.002 | 0 | 0 |

*Cell Contents
      Pearson correlation
      P-Value*

TAD = Trunk angular dispersion, TROM = trunk range of motion, COMROM = center of mass range of motion, Min COM = Minimum of COM, RST = response step time.
