# Supplementary material for: Roles of the prefrontal cortex in learning to time the onset of pre-existing motor programs
Source: PLoS One. 2020 Nov 9;15(11):e0241562. doi: 10.1371/journal.pone.0241562 (PMC7652266; doi:10.1371/journal.pone.0241562)
Supplement: S2 Table — Cell Contents. Pearson correlation. P-Value. TAD = Trunk angular dispersion, TROM = trunk range of motion, COMROM = center of mass range of motion, Min COM = Minimum of COM, RST = response step time. (DOCX) [file pone.0241562.s005.docx]

S2 Table. ΔHbR_AVG_ PFC subregions – Kinematics Correlations.

**PFC subregions**

| **Kinematic**  **variables** | DLPFCL | DLPFCR | VLPFCL | VLPFCR | FPFCL | FPFCR |
| --- | --- | --- | --- | --- | --- | --- |
| TAD | -0.998 | -0.997 | -0.990 | 0.992 | -0.982 | -0.985 |
|  | 0.0001 | 0.0001 | 0.0001 | 0.0001 | 0.0005 | 0.0003 |
|  |  |  |  |  |  |  |
| TROM | -0.999 | -0.997 | -0.990 | -0.993 | -0.988 | -0.990 |
|  | 0.0001 | 0.0001 | 0.0001 | 0.0001 | 0.0002 | 0.0002 |
|  |  |  |  |  |  |  |
| COMROM | -0.996 | -0.997 | -0.994 | -0.994 | -0.981 | -0.986 |
|  | 0.0001 | 0.0001 | 0.0002 | 0.0001 | 0.0005 | 0.0003 |
|  |  |  |  |  |  |  |
| Min COM | 0.994 | 0.993 | 0.992 | 0.992 | 0.976 | 0.980 |
|  | 0.0001 | 0.0001 | 0.0001 | 0.0001 | 0.0009 | 0.0006 |
|  |  |  |  |  |  |  |
| RST | -0.947 | -0.947 | -0.949 | -0.958 | -0.947 | -0.958 |
|  | 0.0004 | 0.0004 | 0.0004 | 0.0003 | 0.0004 | 0.0003 |

*Cell Contents
      Pearson correlation
      P-Value*

TAD = Trunk angular dispersion, TROM = trunk range of motion, COMROM = center of mass range of motion, Min COM = Minimum of COM, RST = response step time.
